# Six-Nine Year Follow-Up of Deep Brain Stimulation for Obsessive-Compulsive Disorder

**DOI:** 10.1371/journal.pone.0167875

**Published:** 2016-12-08

**Authors:** Sarah M. Fayad, Andrew G. Guzick, Adam M. Reid, Dana M. Mason, Agustina Bertone, Kelly D. Foote, Michael S. Okun, Wayne K. Goodman, Herbert E. Ward

**Affiliations:** 1 Department of Psychiatry, College of Medicine, University of Florida, Gainesville, Florida, United States of America; 2 Department of Clinical and Health Psychology, College of Public Health and Health Professions, University of Florida, Gainesville, Florida, United States of America; 3 Department of Psychiatry, Harvard Medical School, Boston, Massachusetts, United States of America; 4 Department of Neurosurgery, College of Medicine, University of Florida, Gainesville, Florida, United States of America; 5 Department of Neurology, College of Medicine, University of Florida, Gainesville, Florida, United States of America; 6 Department of Psychiatry, Mount Sinai School of Medicine, New York, New York, United States of America; University of California San Francisco Department of Neurological Surgery, UNITED STATES

## Abstract

**Objective:**

Deep brain stimulation (DBS) of the ventral capsule/ventral striatum (VC/VS) region has shown promise as a neurosurgical intervention for adults with severe treatment-refractory obsessive-compulsive disorder (OCD). Pilot studies have revealed improvement in obsessive-compulsive symptoms and secondary outcomes following DBS. We sought to establish the long-term safety and effectiveness of DBS of the VC/VS for adults with OCD.

**Materials and Methods:**

A long term follow-up study (73–112 months) was conducted on the six patients who were enrolled in the original National Institute of Mental Health pilot study of DBS for OCD. Qualitative and quantitative data were collected.

**Results:**

Reduction in OCD symptoms mirrored the one-year follow-up data. The same four participants who were treatment responders after one year of treatment showed a consistent OCD response (greater than 35% reduction in Yale Brown Obsessive Compulsive Scale (YBOCS)). Another subject, classified as a non-responder, achieved a 26% reduction in YBOCS score at long term follow-up. The only patient who did not achieve a 25% or greater reduction in YBOCS was no longer receiving active DBS treatment. Secondary outcomes generally matched the one-year follow-up with the exception of depressive symptoms, which significantly increased over the follow-up period. Qualitative feedback indicated that DBS was well tolerated by the subjects.

**Discussion:**

These data indicate that DBS was safe and conferred a long-term benefit in reduction of obsessive-compulsive symptoms. DBS of the VC/VS region did not reveal a sustained response for comorbid depressive symptoms in patients with a primary diagnosis of OCD.

## Introduction

Obsessive-compulsive disorder (OCD) is a chronic and often disabling condition that is more common than previously believed, with an estimated lifetime prevalence of 2.3%.[1–3] The World Health Organization identifies OCD in the top ten most disabling medical conditions, as the syndrome is associated with lost income and decreased quality of life.[4] It is characterized by recurrent, unwanted, intrusive and persistent thoughts, images or impulses (obsessions) and repetitive behaviors or rituals (compulsions). Although potent serotonin reuptake inhibitors (SRIs) are often effective in the treatment of OCD, they have a slow onset of action, requiring 8–10 weeks of treatment to achieve significant reduction in symptoms, and may not be helpful in the most severe OCD cases.[5] Psychotherapeutic interventions have also shown promise, with cognitive behavioral therapy with exposure and response prevention (CBT-ERP) emerging as the most evidence-based psychosocial treatment.[6] During CBT-ERP, patients are challenged to confront feared stimuli without engaging in compulsive behaviors.[7]

Despite recent advances in pharmacological and psychological treatments, some authors have suggested that as many as 20–30% of patients with OCD may remain severely disabled and could be considered candidates for deep brain stimulation (DBS).[8,9] DBS was first described as a potential intervention for debilitating, treatment resistant OCD in a study by Nuttin and colleagues.[10] From 2003 to 2007, researchers at the University of Florida conducted a pilot study funded by the National Institute of Mental Health (NIMH) that examined DBS in six patients with treatment-resistant OCD.[11] This study had a staggered and blinded onset, and observed that four out of six patients were responders and manifested a significant reduction in OCD symptoms with a greater than 35% reduction in Yale Brown Obsessive Compulsive Score (YBOCS) after 12 months of continuous DBS. Given the paucity of long term data on the safety and effectiveness of this procedure in this patient population, we conducted a follow-up investigation on the subjects involved in the original pilot study to assess the long term safety, effectiveness and tolerability of OCD DBS.

Only three other published studies have examined the long term effects of OCD DBS. The initial study by Nuttin and colleagues found that three out of six patients showed significant improvement in obsessive-compulsive symptoms after twenty-one months of anterior limb of the internal capsule region stimulation as evidenced by a YBOCS reduction of 35% or more.[12] Ooms and colleagues assessed quality of life (QOL) and OCD symptom reduction in sixteen patients who had received DBS of the nucleus accumbens for OCD, and found that three to five years after surgery, their cohort experienced improvements in quality of life, OCD symptoms, anxiety, and depressive symptoms.[13] After eight months of treatment, nine of sixteen were classified as responders, while at three to five year follow-up, two additional patients were considered responders using the same 35% reduction in YBOCS criterion.[13] Greenberg and colleagues found in a three year follow-up that DBS of the ventral capsule/ventral striatum (VC/VS) was associated with long-term improvement in obsessive-compulsive symptoms and overall functioning; six out of eight patients demonstrated a 25% or greater reduction in obsessive-compulsive severity, while four out of eight achieved a reduction in YBOCS of at least 35%.[14]

To date, however, no other longitudinal data has been reported that examines effectiveness and safety outcomes beyond three years post-surgery. We present data on long term safety, effectiveness and tolerability of VC/VS DBS in the six patients who were enrolled in a NIMH funded pilot study.

## Materials and Methods

### Participants

Subjects included two males and four females who were initially implanted between October 2003 and January 2007 during a pilot study at the University of Florida (PI: Goodman).[11] The mean age of the six subjects during the follow-up assessment was 44.5 years (range: 33–61).

### Procedure

This study was approved by the University of Florida Gainesville Health Science Center Institutional Review Board. All of the interviews were conducted face-to-face. Two interviews were conducted in person at the University of Florida, and four of these assessments were conducted through video-teleconferencing from the University of Florida to remote locations. All subjects signed informed consents prior to each interview. Assessments and psychometric rating scales were administered to all subjects and information was recorded. Medication and DBS settings at follow-up were obtained through medical records. One subject did not provide consent for medical records and therefore current DBS settings are unknown, though current medications were made available through the interview. The subjects each were given psychometric rating scales, including the YBOCS,[15] the Hamilton Rating Scale for Depression (HAM-D),[16] the Short Form Health Survey (SF-36),[17] and the Montreal Cognitive Assessment (MoCA).[18]

### Statistical Analysis

Descriptive data on the variables of interest are provided throughout the results. An intent-to-treat analysis was conducted using multi-level modeling (MLM) due to its ability to model change in data over time with lower type 1 error than more traditional repeated measures techniques, such as repeated-measures analysis of variance.[19–21] While no power analyses are available for MLM, a general guideline is a sample of 10–30 participants with approximately 5 repeated measures.[22, 23] However, lower sample sizes are adequate when the number of repeated measures is increased,[22, 23] such as in this study in which 32 YBOCS measures were collected across time in 6 patients. The results described below test the fit of a linear growth model to the outcome variable of interest to statistically examine if an increase or decrease in severity was observed. An estimate of the average change during the follow-up period is also provided.

## Results

### Obsessive-Compulsive Symptom Severity

**Fig 1:**
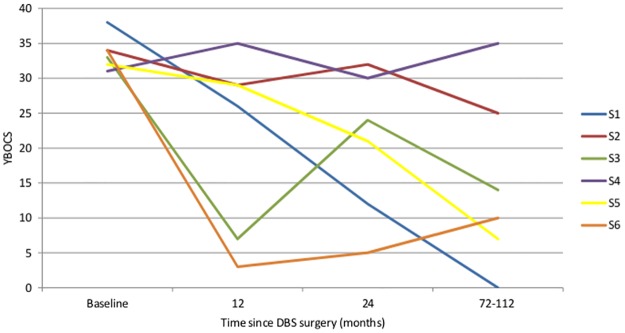
Obsessive-compulsive symptoms across follow-up period. YBOCS = Yale-Brown Obsessive-Compulsive Scale. To improve the clarity of the graph, only data collected at 12 months, 24 months, and at long-term follow-up were included in the figure.

A MLM analysis was fitted for the available monthly data of the primary outcome of the YBOCS during the 114 months of follow-up. A significant linear reduction in symptoms was observed for the group (*F* (1, 162.997) = 15.488, *p* < .001) with an average decrease of 18.5 points on the YBOCS (SD = 14.488) during the follow-up period. The course of obsessive-compulsive severity in the six subjects is displayed in Fig 1. S1, S3, S5, and S6 were all classified as “responders,” or those who experienced at least 35% reduction in their YBOCS scores at the end of follow-up period, while S2 and S4 were classified as “non-responders.” Visual inspection indicates that overall symptoms remitted, although notable fluctuations in symptom severity were observed. Categorical analyses of the data can be observed in Table 1. Echoing the results from the one-year follow-up,[11] the same four participants met criteria for response (≥35% reduction in YBOCS symptoms and YBOCS score ≤ 16). One of the two non-responders experienced a 26% reduction in symptoms, highlighting that with the exception of the subject who was not receiving active DBS, all subjects experienced symptom reduction of at least 25% at long-term follow-up. While four of the subjects had YBOCS scores ≤ 16 at the final follow-up assessment, this did not necessarily indicate continuous symptom remission throughout time, as different numbers of subjects met response criteria at different time points.

**Table 1:** Categorical Responses and Long-Term Follow-Up During DBS for OCD (*n* = 6).

Duration of DBS Activation (months)	Total *n*	<25% YBOCS↓ (n, %)	25–35% YBOCS↓ (n, %)	≥35% YBOCS↓ (n, %)	Severity YBOCS ≤16 (n, %)
1	6	4 (67)	0 (0)	2 (33)	0 (0)
4	6	3 (50)	0 (0)	3 (50)	2 (33)
8	5	2 (20)	0 (0)	3 (60)	3 (60)
12	6	2 (33)	0 (0)	4 (67)	4 (67)
16	6	1 (17)	1 (17)	4 (67)	2 (33)
20	5	2 (40)	0 (0)	3 (60)	2 (40)
24	4	1 (25)	0 (0)	3 (75)	1 (25)
28	5	1 (20)	0 (0)	4 (80)	2 (40)
Long-term follow-up (6 years, 1 month-9 years, 4 months)	6	1 (17)	1 (17)	4 (67)	4 (67)

Note: DBS = Deep Brain Stimulation; OCD = Obsessive-Compulsive Disorder; YBOCS = Yale Brown Obsessive-Compulsive Disorder Scale. Categorical response is shown against duration of DBS activation, which is not the same as time since implantation. Number of nonresponse is indicated in the column labeled <25% reduction on the YBOCS. Number of responders is shown using two different criteria: 25%-35% reduction on the YBOCS and the more stringent definition of ≥35% reduction on the YBOCS. The last column shows the number of individuals who had a YBOCS ≤16 at that time point.

### Secondary Qualitative and Quantitative Data

#### Depressive Symptoms

A MLM analysis was fitted for the available monthly data of the secondary outcome of the HAM-D during the 114 months of follow-up. A significant linear increase in symptoms was observed for the group (*F* (1, 153.267) = 8.637, *p* = .004) with an average increase of 5.833 points (SD = 11.548) during the follow-up period.

**Fig 2:**
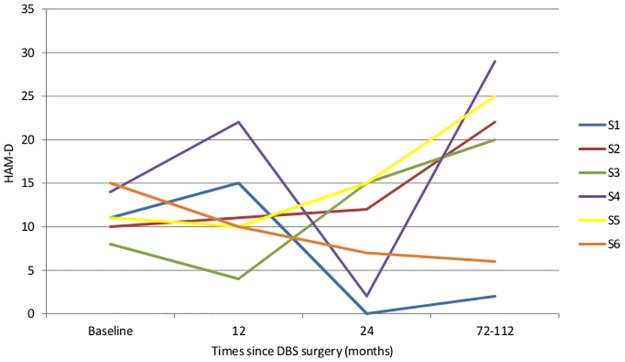
Depressive symptoms across follow-up period. HAM-D = Hamilton Rating Scale for Depression. To improve the clarity of the graph, only data collected at 12 months, 24 months, and at long-term follow-up were included in the figure.

The course of depressive symptom severity in the six subjects is displayed in Fig 2. At the end of the follow-up period, S2 and S3 finished the follow-up period classified as moderately depressed (HAM-D scores between 17 and 23) and S4 and S5 were classified as severely depressed (HAM-D≥23) per the cutoff scores recommended by Zimmerman and colleagues (2013)[24]. Similar to YBOCS scores, visual inspection of this figure suggests frequent fluctuation of depressive symptoms through the course of the follow-up period. As described above, depressive symptoms increased across the follow-up period.

None of the participants began treatment with HAM-D scores above the clinical depression cut-off (score ≥ 17) but four of the six subjects scored in this range at the conclusion of the follow-up period.[24] Both of the DBS non-responders concluded the follow-up period with HAM-D scores above the clinical depression cut-off. See Table 2 for a categorical summary of depressive symptom severity through the course of follow-up.

**Table 2:** HAM-D depression levels across follow-up, per cutoff guidelines.

Duration of DBS Activation (months)	Total *n*	No depression ^a^ n (%)	Mild ^b^ n (%)	Moderate ^c^ n (%)	Severe ^d^ n (%)
Baseline	6	1 (17)	3 (50)	2 (33)	0 (0)
1	6	3 (50)	1 (17)	2 (33)	0 (0)
4	6	3 (50)	0 (0)	1 (17)	2 (33)
8	4	3 (75)	0 (0)	1 (25)	0 (0)
12	6	5 (83)	1 (17)	0 (0)	0 (0)
16	6	3 (50)	2 (33)	1 (17)	0 (0)
20	5	2 (40)	3 (60)	0 (0)	0 (0)
24	4	2 (50)	2 (50)	0 (0)	0 (0)
28	4	2 (50)	1 (25)	0 (0)	1 (25)
Long-term follow-up (6 years, 1 month-9 yrs, 4 months)	6	2 (33)	0 (0)	2 (33)	2 (33)

Note: HAM-D = Hamilton Rating Scale for Depression Clinical cut-offs based of Zimmerman et al. (2013)[24].

^a^ No depression: 0–7;

^b^ mild depression: 8–16;

^c^ moderate depression: 17–23;

^d^ severe depression: ≥24

#### Quality of Life/Functional Impairment

A MLM analysis was fitted for the available monthly data assessing quality of life during the 114 months of follow-up, as measured by the SF-36. The Social Functioning subscale (*F* (1, 49.336) = 12.191, *p* = .001) significantly increased (improved) during the follow-up period, while the General Health subscale (*F* (1, 48.873) = 3.703, *p* = .060) showed improvement trending towards significance. However, the Bodily Pain subscale (*F* (1, 48.010) = 2.980, *p* = .091) showed a decrease trending towards significance. The Physical Functioning, Role-Physical, Vitality, Role-Emotional, and Mental Health subscales did not significantly increase or decrease over the follow-up period.

#### Social Changes

At the time of the follow-up interview, four subjects were married, compared with five at the beginning of the study. Two were unemployed, two reported working part-time, and two said that they had full-time jobs, compared with three who were unemployed and three who worked part-time at the beginning of the study.

#### Neuropsychological Scores

Echoing findings from the original 6 and 12 month follow-ups,[11] there was no indication of neuropsychological impairment when the MoCA was administered at the end of the follow-up period. Specifically, all subjects scored at least 27 on the assessment, exceeding the clinical screening score of 26.[18]

#### DBS Lead Locations and Programming

See Table 3 for a summary of the DBS contacts used for chronic stimulation and the programming settings. Four of six subjects had different settings at 12-month, 30-month, and long-term follow-up. One subject was no longer receiving active DBS and the other subject did not provide consent to obtain medical records for current DBS settings.

**Table 3:** Settings.

Pt	Side	Lateral	AP	Axial	DBS Settings		
					12 month	30 month	Long-term Follow-up
1^a^	R	10.4	16.2	1.7	1- C+, 5 V, 210 μs, 135 Hz	1- C+, 5 V, 210 μs, 135Hz	0-1-C+, 4.5V, 210 μs, 135Hz
	L	6.3	13.7	-3.8	0- C+, 4 V, 210 μs, 135 Hz	0- C+, 4 V, 210 μs, 135 Hz	0-1- C+, 4 V, 210 μs, 135Hz
2	R	10.5	17.3	8.4	2-C+, 3.5V, 210 μs, 135 Hz	1–2-C +, 3.5 V, 210μs, 60 Hz	1+2-, 4 V, 210 μs, 135 Hz
	L	12.8	18.1	9.4	2-C+, 3.5 V, 210 μs, 135 Hz	1–2-C+, 3.5 V, 210 μs, 60 Hz	1+2-, 4 V, 210 μs, 135 Hz
3^a^	R	4.8 (0 Contact)	18.0	-3.8	0-1-C+, 8.5 V, 150 μs, 130 Hz	1- C+, 8.5 V, 150 μs, 130 Hz	0-1-C+ 8.5V, 150 μs, 130 Hz
	L	10.4 (0 Contact)	18.8	-3.8	0-1-C+, 7.5 V, 150 μs, 130 Hz	1- C+, 8 V, 150 μs, 130 Hz	0-1-C+ 8 V, 150 μs, 130 Hz
4	R	8.9	12.4	-2.6	0-1-C+, 6.5 V, 180 μs, 135 Hz	1–2+, 6.5 V, 90 μs, 135 Hz	OFF
	L	13.4	16.0	-2.3	0-1-C+, 6.5 V, 180 μs, 135 Hz	0-1- 2+, 6.0 V, 90 μs, 135 Hz	OFF
5^a^	R	9.2 (0 Contact)	12.2	-1.7	0-1-C+, 2.5 V, 210 μs, 135 Hz	1–0+ 4.0 V, 210 μs, 135 Hz	1-C+, 4.5 V, 150 μs, 130Hz
	L	12.2 (0 Contact)	14.8	4.8	0-1-C+, 2.5 V, 210 μs, 135 Hz	1–0+, 4.0V, 210 μs, 135 Hz	1-C+, 4.5 V, 150 μs, 130 Hz
6^a^	R	9.4	15.9	1.5	1-O+, 3.5V, 90 μs, 135 Hz	1–2-, C+, 3.3 V, 180μs, 135Hz	Unknown
	L	11.2	15.2	.9	1-O+, 3.3V, 90 μs, 135 Hz	1–2-, C+, 3.3 V, 180 μs, 135Hz	Unknown

Note: AP = anteroposterior; DBS = deep brain stimulation; Hz = rate; L = left side; μ = pulse width; R = right side; V = volts; YBOCS = Yale-Brown Obsessive Compulsive Scale; C = case.

^a^ Patients who had clinical response based on YBOCS Criteria (35% reduction). Table shows the settings for the six patients with chronic DBS. The DBS settings show right side, left side, volts, pulse width, and rate. The lateral, anteroposterior, and axial coordinates of the center of the active contact relative to the mid-commissural point are provided.

#### Medication

Three of the subjects (50%) were taking fewer psychotropic medications at long-term follow-up compared with the baseline visit in the pilot study. Two subjects were taking the same number, and one subject went from taking one psychotropic medication to two. See Table 4 for a summary of psychotropic medications at baseline, 30-month follow-up and long-term follow-up.

**Table 4:** Psychotropic Medications.

Participant #	Baseline	30 month follow-up	Long-term follow-up
1^a^	Clonazepam 0.5 mg bid	none	Duloxetine 30 mg daily
2^b^	Escitalopram 30 mg daily	Escitalopram 30 mg daily	Trazodone 100 mg qhs Sertraline 200 mg
3^b^	Fluvoxamine 100 mg tid Lamotrigine 150 mg tid Clonazepam 0.5 mg bid Mirtazapine 60 mg qhs	Fluvoxamine 400 mg daily	Fluvoxamine 400 mg daily
		Aripirazole 10 mg daily Modafinil 400 mg daily	Aripiprazole 10 mg daily Buspirone 15 mg bid Modafinil 600 mg daily
4^c^	None	Aripiprazole 20 mg daily	None
5^c^	Escitalopram 80 mg daily Clonazepam 0.5 mg daily Bupropion 300 mg daily Risperidone 1 mg qhs	Escitalopram 25 mg daily Clonazepam 1 mg tid	Escitalopram 50 mg daily
			Clonazepam 0.5 mg qid Gabapentin 600 mg bid
6^a^	Topirimate 25 mg qhs Lamotrigine 200 mg qhs Fluoxetine 80 mg daily Lorazepam 2 mg qid	Topirimate 25 mg qhs Quetiapine 300 mg qhs Fluoxetine 60 mg daily Clonazepam 2mg qhs	Fluoxetine 40 mg daily Diazepam bid

Note:

^a^ not clinically depressed,

^b^ moderate depression,

^c^ severe depression. Table shows the medication prescribed at baseline, 30-month follow-up, and long-term follow-up for the six patients with chronic OCD who received DBS.

#### Adverse events

Only one serious adverse event was reported. One subject was admitted to an inpatient psychiatric facility following a DBS settings adjustment within the first year post-implantation for worsening OCD.[11] Once the settings were appropriately readjusted the patient improved and was discharged.

Four subjects endorsed sleep disturbances, although the specific nature of the problem varied from patient to patient. One reported insomnia on the night of implanted pulse generator replacement, another reported difficulty when voltage was adjusted and too high, and another reported sleeping too much and a recurrence of OCD symptoms when the IPG was depleted. The final patient reported general insomnia unrelated to DBS. Other anecdotal adverse events include shooting tingling on the right side of body, metal taste and jaw clenching, and mild skin redness and burning on head and chest. All these adverse events were episodic, with the exception of one patient who reported slightly increased fatigue following DBS.

#### Qualitative Feedback

The five subjects whose YBOCS scores improved at least 25% subjectively reported that their symptoms had improved since starting DBS. The four responders all reported that they would elect to receive DBS again if given the choice. The subject who experienced a 26% improvement stated they would likely not choose DBS again due to discomfort of the stereotactic device during implantation and due to their desire for greater benefit in terms of obsessive-compulsive symptom relief. The patient whose DBS was turned off shortly after the beginning of the study stated they would choose DBS again, depending on the cost of the procedure.

During the interview, subjects were given an opportunity to voice any negative experiences to receiving DBS. There were a few notable complaints. One subject wished his/her other physicians knew more about DBS, and felt they may have been treated differently by medical professionals due to their interest with the procedure. Another subject reported feeling self-conscious about surgery-related scars and battery bulge, while another was surprised that there were no cosmetic issues with DBS. One subject noted that finding optimal programming settings for DBS can be problematic and expensive.

Finally, we questioned subjects about the perceived benefits and drawbacks of rechargeable IPGs versus non-rechargeable IPGs. The three patients who received rechargeable IPG’s expressed a preference for them, as frequent surgeries are necessary with non-rechargeable batteries (every 6–12 months with non-rechargeable vs. every 7–8 years with rechargeable). Patients noted the limitations of the rechargeable batteries as well, specifically that they had to be recharged for about 30 minutes per day. One patient believed that the rechargeable batteries were slightly less effective, but the lack of frequent surgery made up for any quality of life deficits.

## Discussion

There has been very little long term data available regarding safety and effectiveness of OCD DBS. This long term follow-up provides valuable information supporting both safety and effectiveness in this patient population over time (up to nine years and four months after implantation of DBS). There was significant reduction in OCD symptoms (*p* < 0.001) across this pilot group, with an average of an 18 point reduction in YBOCS scores. Four out of six subjects met criteria for response (greater than 35% reduction in YBOCS), with an additional subject having a reduction of 26% in YBOCS score. These results were very similar to previous data published by Greenberg and colleagues in which six of eight subjects achieved a 25% or greater reduction in YBOCS compared to baseline.[14] The only subject who did not demonstrate reduction in YBOCS score had the device turned off. Across the entire follow-up period, there was only one serious adverse event reported, which occurred during the first 12 months (i.e., psychiatric hospitalization due to a DBS setting adjustment that resolved with a programming session). Other side effects were minor, usually related to setting or battery changes, and easily remediated with adjustments in settings. The safety data support OCD DBS as a reasonably safe intervention.

While there was a significant reduction in obsessive compulsive symptoms over time among the majority of patients, there were notable fluctuations in severity of both OCD and depression over time (Figs 1 and 2). These fluctuations did not correspond with battery depletion or changes. It was unclear what led to fluctuations in symptoms other than daily life stressors. It should be noted that fluctuations in obsessive-compulsive and depressive symptoms have been well-documented in naturalistic follow-up studies.[25, 26]

One notable finding in this study was that there was a significant increase in depressive symptoms (*p* < 0.01), with an average increase of approximately six points on the HAM-D rating scale. At baseline, only two of six subjects met criteria for a moderate depression (Table 2), with three subjects meeting criteria for mild depression and one with no depression. At the end of the long term study, two patients met criteria for moderate depression and two met criteria for severe depression, while two had no depressive symptoms based on the HAM-D. There have been subsequent DBS studies published addressing major depressive disorder. DBS was placed in the same anatomical region as our study,[27] partially based the target selection and response to comorbid depression in OCD. These studies showed initial improvement in depression, but larger recent studies have not revealed a benefit. These findings collectively suggest that this area of the brain may not be optimal for long term treatment resistant depression (without OCD), though there have been limitations to interpretation of these data including a small sample size and a lack of knowledge on the optimal targets and settings for depression DBS. Medication adjustments as would be expected, took place over the course of the follow-up of our patients (see Table 4). However, in the two patients who were not clinically depressed at long term follow-up, one had switched from a benzodiazepine to low dose duloxetine, and the other had a significant reduction in the number medications prescribed. Of the four subjects who experienced a worsening in depression over the course of the study, only one had an overall reduction in antidepressant medication, while the other subjects’ medication regimens stayed the same, were augmented, or were increased. Thus, it did not appear that medication reduction led to the increase in depressive symptoms. Over a six to nine year follow-up, any number of stressful life circumstances may have exacerbated depressive symptoms, especially in a cohort at high risk for developing mood disorders.[28]

In terms of disability, social functioning improved significantly, general health showed slight improvement and bodily pain worsened. None of the subjects reported chronic pain as a result of the DBS at the follow-up interview, therefore we propose the increase in self-reported bodily pain may be tied to normal aging or to elevated depressive symptomology. The remainder of subscales, including physical functioning, role-physical, vitality, role-emotional and mental health subscales did not show significant changes over the long term. There were no negative cognitive sequelae.

From our qualitative data, all subjects but one indicated they would choose to do DBS again. The subject who indicated he would not elect to do DBS again related it to discomfort with the stereotactic device in surgery, despite a 26% reduction in OCD symptoms. Subjects who received rechargeable batteries preferred these to non-rechargeable batteries, citing improved quality of life.

There are several notable limitations to this long term follow-up study. It was not controlled and subjects had medication and DBS setting adjustments throughout the course of the long-term follow-up. It is therefore difficult to attribute all changes to DBS alone in this context. In addition, the sample size was overall quite small and larger numbers of subjects will be required to fully evaluate the long term effects of DBS of the VC/VS on OCD and comorbid depression.

VC/VS DBS was safe and conferred a long term benefit in reduction of OCD symptoms. Nearly all subjects receiving the therapy would elect to receive it again. Quality of life was improved quantitatively and qualitatively, though depressive symptoms significantly increased over the follow-up period. All subjects who received rechargeable IPG’s voiced a preference over traditional IPG’s citing the frequent need for replacement surgery. Patient selection is critically important for OCD DBS and should be conducted in expert interdisciplinary centers with experienced psychiatrists managing neuropsychiatric complications which can emerge post-DBS.
